# Multiplicity of Mesenchymal Stromal Cells: Finding the Right Route to Therapy

**DOI:** 10.3389/fimmu.2019.01112

**Published:** 2019-05-16

**Authors:** Alison Wilson, Margeaux Hodgson-Garms, Jessica E. Frith, Paul Genever

**Affiliations:** ^1^Department of Biology, University of York, York, United Kingdom; ^2^Materials Science and Engineering, Monash University, Clayton, VIC, Australia

**Keywords:** mesenchymal stromal cell, heterogeneity, cell subpopulations, cell-based therapy, single cell technologies

## Abstract

Over the last decade, the acceleration in the clinical use of mesenchymal stromal cells (MSCs) has been nothing short of spectacular. Perhaps most surprising is how little we know about the “MSC product.” Although MSCs are being delivered to patients at an alarming rate, the regulatory requirements for MSC therapies (for example in terms of quality assurance and quality control) are nowhere near the expectations of traditional pharmaceuticals. That said, the standards that define a chemical compound or purified recombinant protein cannot be applied with the same stringency to a cell-based therapy. Biological processes are dynamic, adaptive and variable. Heterogeneity will always exist or emerge within even the most rigorously sorted clonal cell populations. With MSCs, perhaps more so than any other therapeutic cell, heterogeneity pervades at multiple levels, from the sample source to the single cell. The research and clinical communities collectively need to recognize and take steps to address this troublesome truth, to ensure that the promise of MSC-based therapies is fulfilled.

## Introduction

The term “MSCs” is used to describe a heterogeneous population of stromal cells, the exact nature and composition of which remains the subject of much debate. They are often characterized using criteria proposed by the International Society for Cell Therapy (ISCT) as plastic-adherent cells, expressing a distinct set of surface antigens and with the ability to differentiate *in vitro* into osteogenic, adipogenic, and chondrogenic lineages (1). This minimal definition, however, is far from definitive. MSCs exhibit unique immunomodulatory properties, support the hematopoietic niche and participate in tissue regeneration through diverse biological activities including engraftment-independent paracrine signaling. Though initially described and sourced from bone marrow we are now able to isolate MSC-like cells from a variety of tissues including adipose tissue, dental pulp, placenta, umbilical cord, and umbilical cord blood.

**Figure 1:**
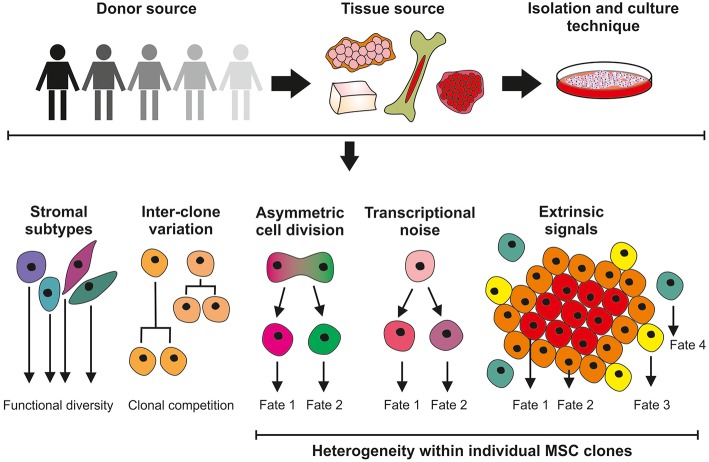
Sources of MSC heterogeneity; considerations for the clinical application of culture-expanded MSCs. Significant variation exists in MSC cultures isolated from different donors and different tissue sites. Unrefined and non-standardized isolation and culture techniques do not select for homogeneous cell populations and are likely to give rise to a mixture of stromal cell with different functions. Differences in the growth properties of MSC clones can result in cultures being dominated by the faster-growing lines. Further levels of heterogeneity can be introduced within MSC clones through asymmetric cell division and the effects of stochastic transcriptional noise, generating cells with modified phenotypes. MSC properties will also be determined by, for example, proximity to neighboring cells and extrinsic signaling factors.

Although MSCs first appeared in the clinic in 1995 (2) and have since become one of the most clinically studied cell therapy platforms worldwide (3) many fundamental aspects of MSC biology remain undetermined; primarily a direct consequence of the pervasive heterogeneity that manifests itself between MSC donors, tissue sources, culture methods and individual cells within a clonal population. Furthermore, MSCs exhibit a remarkable level of plasticity over time and when presented with different microenvironments (4, 5). MSC multiplicity, and a lack of consensus in the scientific community, complicates MSC characterization and their translation into the clinic. This review will consider the multilevel origins of heterogeneity in MSCs (see Figure 1) and how we should be doing more to identify, track and quantify heterogeneity in MSCs to help determine its biological importance and impact in *in vitro* and *in vivo* contexts.

## Change Is the Only Constant (*Heraclitus, 535–475 BC*)

MSC heterogeneity has certainly obscured our understanding of MSC biology and, correctly, prompted calls to re-evaluate the use of MSCs in therapy (6–10). However, the origins of heterogeneity are complex, fascinating and a constant theme in biology. It is clear from other work, particularly in microbial systems, that heterogeneity arising in genetically identical populations can have a positive impact on overall population fitness (11–14). Stochastic fluctuations in gene expression, or “noise,” can lead to phenotypic variability in clonal cell populations (11, 15) and “bet hedging” can confer survival advantages on individual cells within mixed communities when faced with environmental change (16, 17). It has been proposed that stochastic non-genetic variations (i.e., those not caused by genetic mutations) contribute to the evolution of tumors using bet hedging-like strategies (18–20) and the dynamic switching between subtly different phenotypes has been shown to influence cell fate in different adult and embryonic stem cell populations (21–23). Gene expression noise in MSCs is also likely to give rise to individual cells with different characteristics and therefore influence the aggregate function of the population. It is also clear that MSC heterogeneity is due at least in part to the existence of different subpopulations with distinct expression profiles and functional properties (24–26). It has not been determined if discrete stromal subpopulations evolve through stochastic or deterministic means, but many appear to possess properties that support general tissue maintenance [for example, immune control, vascular remodeling, hematopoiesis (25)] that are unrelated to stem cell function. Therefore, the umbrella “MSC” descriptor may actually cover a range of related but distinct cell types that are yet to be fully defined.

## Impact of Donor- and Tissue-Dependent MSC Heterogeneity

Cells that currently meet this broad MSC descriptor have been identified in virtually all post-natal organs and tissues (27) and while bone marrow derived MSCs (BM-MSC) are still considered the gold standard, MSCs are now frequently also isolated from adipose tissue (AT-MSCs) and umbilical cord or cord blood (UC/UCB-MSCs) (28–33). There are well-documented disparities in proliferation, differentiation potential, surface markers, transcriptional, and proteomic profile of MSCs from different sources (34–36); an overarching consensus is hard to come by. For example, prevailing MSC characteristics such as tri-lineage differentiation potential present contradictory evidence in terms of lineage preference and full tri-lineage capacity (29, 30, 32, 37). Even when derived from the same tissue of origin, MSCs demonstrate prodigious donor-to-donor variation. This may be a factor of donor health influencing MSC availability and function (38, 39). Donor age can also affect self-renewal capacity and differentiation potential, which have been reported to decline in older donors (40–43). However, differences are also apparent in healthy donors of a similar age in proliferation rate, differentiation capacity, and ultimate clinical utility (44) leading to a further addition of complexity when directly comparing samples. It is tempting to speculate that MSC heterogeneity mirrors the diversity of environments from which they may be isolated, the reality is however that our understanding of MSCs *in vivo* is still in its infancy (8).

The multiplicity of MSCs and the absence of a meaningful consensus on definitions and characterization parameters makes comparing studies within the field difficult and translating them into clinical practice even more so. Because heterogeneity is seldom accounted for, and unique cell populations used in individual research projects are rarely fully defined, many studies are not only difficult to reproduce but difficult to evaluate for comparability and impact within the field. Incomplete knowledge of the characteristics of MSCs *in vivo* and how these will relate to clinical outcomes further exacerbate the problem when considering quality control requirements for MSCs as therapeutic agents. Changes in the source materials of clinical products, e.g., a different donor, prompt regulatory authorities to require re-characterization and evidence of “comparability.” In the event that comparability could not be demonstrated, product from the original and subsequent sources would be considered to be essentially different products. Thus, during clinical development, data on early product iterations could be invalidated, and post-authorization could, in the worst-case scenario, require re-authorization. In conjunction with the need for adequate cell numbers, this represents a major challenge to the acceptance of cell-based therapies as mainstream treatments; the options of extended culture or multiple donors each imply unavoidable heterogeneity. Consequently the manufacture of MSC products using processes that rely on a continuous supply of new tissue donations run the significant risk of supply constraint, interruption, and inconsistencies (10).

## *In vitro* Expansion and MSC Heterogeneity

A typical bone marrow aspirate contains just 0.01–0.001% MSCs (45) and trials for the regeneration of bone and cartilage tissue commonly use in the order of 10 million cells. The need for high levels of culture expansion adds to the challenge of generating an MSC population that retains the ability to differentiate effectively or secrete the appropriate biomolecules to induce a beneficial paracrine response. Banfi et al. investigated the growth kinetics and differentiation potential of MSCs, using fresh isolates from different donors through to passage five, and showed a dramatic decrease in MSC functionality over time (46). MSCs from the same donor and same source (iliac crest marrow aspirate) isolated at different timepoints over a period of 6 months also show significant variation in growth rates (44). Other studies have confirmed this loss of MSC function, demonstrating reduced proliferation, colony-forming (CFU-f) efficiency, telomere length and differentiation capacity with increasing time in culture (4, 40, 47). With the mounting interest in the use of MSCs for their paracrine effect it is also noteworthy that the secreted output of MSCs has been shown to differ with number of passages (48). This reduction in therapeutic potency at the population level can mask changes within clonal MSCs. Schellenberg et al. assessed MSC clones following expansion and observed a continual decrease in CFU-f efficiency and differentiation capacity over time (49). Earlier analyses identified a complex hierarchy of MSC clones at varying stages of potency (50), so it may be that the diminishing clonal potential observed during MSC expansion is driven by subsets of cells reaching their proliferative limit or by entering the hierarchy of different stages through which cells pass during differentiation. Subsequent studies to track individual clones from MSC explant cultures showed that clonal complexity decreased markedly over 12 passages resulting in the clonal selection of a few dominant MSC clones (51).

Given the impact that culture expansion has on MSC fate, the *in vitro* environment and its influence on MSC properties is worth considering. In the majority of research laboratories, MSCs are expanded as a monolayer using standard tissue culture flasks with a plasma-treated polystyrene surface and medium containing fetal bovine serum. Surprisingly, given the detrimental effects on MSC proliferation, differentiation and paracrine activity of these basic methods, the industrial expansion of MSCs for clinical applications often still retains the same basic features (52). Scale-up can be achieved through the use of multilayered cell culture flasks (cell factories) or culture vessels specifically tailored for use with closed-box and automated systems. More advanced systems use roller bottles, hollow-fiber or stirred tank bioreactors [reviewed by (53)]. A major problem with this approach is that that these *in vitro* conditions are very different from the *in vivo* MSC microenvironment, lacking much of the complexity in terms of matrix composition, geometry, mechanical properties and interactions with other cell types. All of these microenvironmental factors are interpreted by the cell and have been shown to impact upon their behavior (54–59). At its worst, the non-physiological conditions of typical cell cultures can cause mutations or cellular defects (60) but even the best-case scenario results in cells whose behavior is markedly changed. Together, this results in loss of potential from the whole population, but MSC heterogeneity may also be driven by cells responding to local changes in the microenvironment, such as through poorly controlled substrate properties or local changes in oxygen and nutrient concentration driven by the static nature of the setup (61).

It is clear that the requirement for extended *in vitro* expansion is a major contributor to the heterogeneity of MSC populations. A deeper understanding of the impacts of different environmental cues and the mechanisms by which they drive change, will be integral to the development of technologies for the large-scale production of quality MSC populations for clinical use.

## Clinical Experience and Regulatory Considerations Related to Heterogeneous Cell Therapy

MSC heterogeneity is multifactorial and functionally influential. Nonetheless the clinical application of MSCs does not appear to take this into account, with a selection of recent trial publications suggesting a comparatively limited assessment of cellular phenotype (Table 1). The criteria established for MSCs by the ISCT (1) are sometimes referenced in these studies but not necessarily met. It is of course possible that additional criteria were specified during manufacture but not published, however publication of more detail would increase our understanding of the MSC phenotypes in clinical use.

**Table 1:** Sample characterization and release criteria reported in clinical trials using MSCs.

**Phase**	**Indication**	**Tissue**	**Source**	**Characterization**	**Stated release criteria**	**Notes**	**References**
I	Myocardial infarction	Bone Marrow	Allo		Positive: CD105, CD166 limits NS Negative: CD45 limits NS	“Provacel”—became Prochymal	(62)
I	Crohn's disease	Bone Marrow	Auto	HLA II (DR), CD73, CD90, CD31, CD34, CD45, CD80, CD105	CD73, CD90, and CD105 >90%		(63)
I	Graft vs. Host Disease	Bone Marrow	Allo		Positive: CD73, CD90, CD105 limit NS, Negative: CD14, CD34, CD45 limit NS		(64)
II	Graft vs. Host Disease	Bone Marrow	Allo	CD105, CD59, CD73, CD90, CD31, CD34, CD14, CD45, HLA-DR, FSP	NS		(65)
II	Multiple sclerosis	Bone Marrow	Auto	CD90, CD90, CD31, CD34, CD45	ISCT criteria	Phenotypic analysis not consistent with ISCT	(66)
I	Osteoarthritis (knee)	Bone Marrow	Auto	Positive for CD90, CD105, CD106, CD166, KDR (VEGFR2). Negative for CD34, CD45, HLA-DR	ISCT criteria	Data not presented	(67)
I	Transplant rejection	Bone Marrow	Auto	HLA II (DR), CD73, CD90, CD31, CD34, CD45, CD80, CD105	CD73, CD90, CD105 >90%		(68)
II	Kidney structure/function	Bone Marrow	Auto	HLA II (DR), CD73, CD90, CD31, CD34, CD45, CD80, CD105	CD73, CD90, CD105 >90%	Trial design, study not reported	(69)
I	Graft vs. Host Disease	Bone Marrow	Allo		CD73, CD90, CD105 >80% CD14, CD34, CD45 <10%		(70)
II	Crohn's disease	Bone Marrow	Allo		ISCT criteria	Data not presented	(71)
II	Multiple sclerosis	Bone Marrow	Auto		Positive: CD90, CD73, CD44 limits NS. Negative: CD34, CD45 limits NS		(72)
II	Myocardial infarction	Bone Marrow	Auto		Positive: CD73, CD105 >90%. Negative: CD14, CD34, CD45 <3%		(73)
I	Acute Respiratory Distress Syndrome	Bone Marrow	Allo			FC performed but no data presented	(74)
I	Osteoarthritis (knee)	Adipose	Auto	CD73, CD90, CD105, CD14, CD31, CD34, CD45, CD80, IgG1	CD14, CD45 <2% CD34 <10% CD73, CD90 >90%, CD105 >80%		(75)
I/IIa	Meniscus	Bone Marrow	Auto		Positive: CD90, CD105 >80%. Negative: CD34, CD45 <10%		(76)

Basic requirements for all biological medicines include the necessity to define the identity, the purity and the potency of the product. The developers of cell-based medicinal products must define the “active substance”; the cell type on which the therapeutic action of the product depends. Specification limits must be established for unique identification of the active substance within the product and for quantitation of its purity. Other phenotypes present, for example those arising from a tissue biopsy or culture contaminant, and non-viable cells, are generally regarded as impurities. These impurities should be reduced as far as possible and their content in the finished product limited and defined by specifications. Cellular impurities aside, major regulatory authorities do not always require cell-based medicinal products to consist of a pure population of cells. One of the first authorized cellular therapies was the immunotherapy Provenge (Dendreon Inc), approved by the US Food and Drug Administration (FDA) in 2010 for treatment of certain prostate cancers. Provenge contains autologous peripheral blood mononuclear cells (PBMC), which are cultured with PAP-GM-CSF, a fusion protein combining granulocyte-macrophage colony-stimulating factor (GM-CSF) with a prostate cancer antigen (prostatic acid phosphatase, PAP). Antigen-presenting cells within the PBMC fraction are activated by the fusion protein, providing a tumor-directed action. The exact composition of the Provenge dose varies depending on the cellular composition of each patient's leukapheresis sample, but may contain, amongst others, T and B lymphocytes and natural killer cells so the therapy is inherently heterogeneous (77, 78). In 2015 the European Union (EU) authorized its first stem cell-based product, Holoclar (Chiesi Farmaceutici SPA, Italy). Holoclar is a population of cultured autologous human corneal epithelial cells containing limbal stem cells (LSCs) intended for treatment of ocular burns. The active substance contains only approximately 3.5% of p63bright LSCs, in a mixed population with transient amplifying meroclones and paraclones and terminally differentiated corneal epithelial cells (79). The extensive heterogeneity of the overall product, which arises from the inherent cellular variation in the patient's biopsy, was justified by evidence of relevant supportive properties provided by the non-stem majority population; these were therefore not considered to be cellular impurities (80).

In 2016, the EU approved Strimvelis [Orchard Therapeutics (Netherlands) BV], a gene therapy for treatment of adenosine deaminase (ADA) severe combined immunodeficiency (ADA-SCID), in which autologous CD34+ hematopoietic stem cells (HSCs) were transduced with ADA cDNA to provide the missing gene sequence. The active substance of Strimvelis includes not only the transduced CD34+ cells, but also the non-genetically modified CD34+ fraction, based on the fact that HSC transplantation is itself a standard treatment for ADA-SCID (81) These examples provide illustrations of the general acceptability, where justified, of heterogeneous cell populations within authorized cellular therapies. In the latter two cases, the heterogeneity specifically contributes to the overall clinical effect of the product and is not merely a consequence of the manufacturing process. The complexity associated with using fundamentally variable starting materials which are then processed, inducing further heterogeneity, implies that the purity of most cell-based products will be challenging to define. The regulators' expectation of quantitation of the population being administered in terms of identity and purity (82, 83) will be difficult to achieve definitively; it is probably more reasonable to demonstrate a degree of reproducibility across product batches and to relate the composition of each batch to those used in clinical trials than to provide exact percentages of each minor cellular component (84). The identification of relevant mechanisms of action will be of crucial importance in determining the acceptability of a degree of heterogeneity, since MSC activity in a specific clinical application should help inform selection of an ideal MSC population, whether this may be a heterogeneous preparation or a specified subset.

The inevitability of MSC heterogeneity and the consequences of culture expansion for the production of cell therapies, discussed earlier, raise key questions for developers of regenerative medicines. Whilst, as illustrated above, there is no obligation to demonstrate that a product contains only the specific cell type of interest, the challenges of definition and identification are accentuated when considering MSCs. The apparent absence of major concerns around cellular heterogeneity in whole organ and HSC transplantation is sometimes highlighted as support for a less rigorous approach to the characterization and control of cell-based therapies. However, acceptance of heterogeneity in these situations may be due in part to the fact that organ and HSC transplants are procedures which are considered to fall within the practice of medicine rather than items externally regulated as medicinal products.

## Future Perspectives: Embracing Change

In order to advance the clinical utility of MSCs, it is essential that strategies to quantify heterogeneity are agreed. As a starting point, it is important to define the biological properties of the different stromal cell types within a mixed population. It is likely that stem-cell and non-stem-cell fractions are co-extracted using current protocols for MSC isolation. For regenerative therapies, it would seem logical that the stem-cell component is the essential active ingredient, however non-differentiating stromal cells could play important supporting roles, for example in immune control; precisely why we need a full biological understanding that relates to mechanism of action. This can be achieved by exploiting techniques suitable for phenotyping individual cells, including flow cytometry, electrophysiology, microscopy (in various forms), image/morphometric analysis, lineage tracing, and powerful new single cell-omic technologies. Effective strategies will be required to ensure data are integrated, interpreted correctly and shared. The key to clinical translation will be to develop the most appropriate non-destructive biomarker identification techniques that provide functional discrimination. Reliable subtype-specific biomarkers will also support the development of treatments to target MSCs *in situ*, potentially negating the need for culture expansion. Alongside these, improved methods for MSC expansion that retain, or even promote selection of the desired MSC properties will be essential for the production of MSC products with a more defined set of characteristics and high therapeutic efficacy. Such technologies will likely incorporate biophysical as well as biochemical cues and provide platforms for scale-up culture in bioreactors. With the role of the paracrine effect of MSCs coming to the fore (85), therapies based on the MSC secretome or MSC-derived extracellular vesicles (EVs) may emerge to complement the MSC therapeutic toolkit. However, different MSC populations (or cells within that population) are still likely to produce different secretomes and so many of the fundamental challenges relating to MSC heterogeneity will remain.

Given the challenges associated with providing consistency in an MSC product from multiple tissue isolates, the generation of MSCs from pluripotent stem cell populations has garnered interest (86–92). The expansion capability of pluripotent cells means that a single clonal population can potentially be manufactured and subsequently differentiated into a virtually limitless supply of MSCs. This type of platform relieves the need for continuous tissue donations, simplifies the subject of donor-donor variation and bypasses many of the sources of MSC heterogeneity that arise when working with *ex vivo* cells. Induced pluripotent stem cells (iPSC)-derived MSCs offer the potential for large-scale production of more homogenous, off-the-shelf products with limited batch-to-batch variation that could deliver more consistent clinical outcomes. The first phase I clinical trial using iPSC-derived MSCs was completed in 2018 with promising results from Cynata Therapeutics's lead Cymerus™CYP-001 product for the treatment of graft vs. host disease (93), although the full findings have not yet been published. While the clinical use of iPSC-MSCs holds promise, an effective comparison of pluripotent cell-derived MSCs to their adult tissue counterparts is required, with appropriate safety profiling. Clonal immortalized MSC lines (both iPSC-derived and genetically modified adult MSCs) may also be developed for bulk harvesting of secreted products, proteins, and EV cargoes, which could ultimately dispense with the need for the transplantation of MSCs as a whole-cell product, however the issue of stochastic heterogeneity arising in clonal cell populations will always persist.

MSCs can offer widespread therapeutic benefits but we must balance enthusiastic demands for clinical progress against the need for better mechanistic understanding. Unraveling MSC multiplicity is the essential first step in that process.

## Author Contributions

AW, MH-G, JF, and PG wrote sections of the manuscript. All authors contributed to manuscript revision, read and approved the submitted version.

### Conflict of Interest Statement

JF and MH-G acknowledge funding from Cynata Therapeutics. The remaining authors declare that the research was conducted in the absence of any commercial or financial relationships that could be construed as a potential conflict of interest.
